# Surface antigen profiles of leukocytes and melanoma cells in lymph node metastases are associated with survival in AJCC stage III melanoma patients

**DOI:** 10.1007/s10585-014-9636-7

**Published:** 2014-01-17

**Authors:** Kimberley L. Kaufman, Swetlana Mactier, Nicola J. Armstrong, Duthika Mallawaaratchy, Scott N. Byrne, Lauren E. Haydu, Valerie Jakrot, John F. Thompson, Graham J. Mann, Richard A. Scolyer, Richard I. Christopherson

**Affiliations:** 1 School of Molecular Bioscience, University of Sydney, Sydney, NSW 2006 Australia; 2 Garvan Institute of Medical Research, Darlinghurst, NSW 2010 Australia; 3 School of Mathematics and Statistics and Prince of Wales Clinical School, University of New South Wales, Kensington, NSW 2052 Australia; 4 Discipline of Infectious Diseases and Immunology Sydney Medical School, University of Sydney, Sydney, NSW 2006 Australia; 5 Discipline of Dermatology, Bosch Institute, Faculty of Medicine, Sydney Medical School, Sydney, NSW 2006 Australia; 6 Melanoma Institute Australia, North Sydney, NSW 2060 Australia; 7 Discipline of Surgery, Sydney Medical School, The University of Sydney, Sydney, NSW 2006 Australia; 8 Westmead Institute of Cancer Research, The University of Sydney at Westmead Millennium Institute, Westmead, NSW 2145 Australia; 9 Department of Tissue Pathology and Diagnostic Oncology, Royal Prince Alfred Hospital, Camperdown, NSW 2050 Australia; 10 Discipline of Pathology, Sydney Medical School, The University of Sydney, Sydney, NSW 2006 Australia

**Keywords:** Antibody microarray, Metastatic melanoma, CD antigen, Survival, Prognosis

## Abstract

There is an urgent need to identify more accurate prognostic biomarkers in melanoma patients, particularly in those with metastatic disease. This study aimed to identify melanoma and leukocyte surface antigens predictive of survival in a prospective series of AJCC stage IIIb/c melanoma patients (*n* = 29). Live cell suspensions were prepared from melanoma metastases within lymph nodes (LN). The suspensions were immuno-magnetically separated into CD45^+^ (leukocyte) and CD45^−^ (non-hematopoietic, enriched melanoma cell) fractions. Surface antigens on CD45^−^ and CD45^+^ cell populations were profiled using DotScan™ microarrays (Medsaic Pty. Ltd.) and showed differential abundance levels for 52 and 78 antigens respectively. Associations of the surface profiles with clinicopathologic and outcome data (median follow-up 35.4 months post LN resection) were sought using univariate (log-rank test) and multivariate (Wald’s test; modelled with patient’s age, gender and AJCC staging at LN recurrence) survival models. CD9 (*p* = 0.036), CD39 (*p* = 0.004) and CD55 (*p* = 0.005) on CD45^+^ leukocytes were independently associated with distant metastasis-free survival using multivariate analysis. Leukocytes with high CD39 levels were also significantly associated with increased overall survival (OS) in multivariate analysis (*p* = 0.016). LNs containing leukocytes expressing CD11b (*p* = 0.025), CD49d (*p* = 0.043) and CD79b (*p* = 0.044) were associated with reduced OS on univariate analysis. For enriched melanoma cells (CD45^−^ cell populations), 11 surface antigens were significantly correlated with the disease-free interval (DFI) between diagnosis of culprit primary melanoma and LN metastasis resection. Nine antigens on CD45^+^ leukocytes also correlated with DFI. Following validation in independent datasets, surface markers identified here should enable more accurate determination of prognosis in stage III melanoma patients and provide better risk stratification of patients entering clinical trials.

**Electronic supplementary material:**

The online version of this article (doi:10.1007/s10585-014-9636-7) contains supplementary material, which is available to authorized users.

## Introduction

Melanoma is a major cause of morbidity and mortality in many Western countries and its incidence is increasing [1]. In these populations, melanoma is one of the most common cancers amongst young adults and is a major public health problem [2, 3]. Compared with other cancers, melanoma has a propensity for metastasis early in disease progression, even from thin (early stage) primary tumors [4]. Nevertheless, when detected and treated early, melanoma has a high cure rate. Mortality in melanoma is usually a consequence of metastatic spread. A detailed melanoma staging system was developed by the American Joint Committee on Cancer (AJCC) to stratify patients into prognostic groups, taking into account the principal prognostic factors including primary tumor characteristics (such as tumor thickness, ulceration, mitotic rate) and the presence, number and size of loco-regional and distant metastases [5]. However, outcomes for patients within individual AJCC stage sub-categories can be vastly different, particularly for those with metastatic disease. For example, 50 % of patients who present with clinically palpable regional lymph node (LN) metastases (AJCC stage IIIc disease) will be dead within 2 years. However, 50 % of those who are still alive at 2 years will live for more than 20 years. Identification of parameters and biomarkers that can collectively predict prognosis for AJCC stage III patients would facilitate more accurate risk stratification, rational selection of patients who may benefit from adjuvant therapies, and development of screening and follow up protocols.

Many studies have sought to identify protein biomarkers, including surface antigens that can be utilized for prognosis of primary melanoma [6, 7]. Whilst some biomarkers correlate with melanoma progression and survival, few have shown significance independent of other well known and more easily measured prognostic parameters (such as tumor thickness) and even less have been validated in independent datasets [8–20]. An extensive immuno-phenotype (surface profile) using antibody microarrays may identify disease signatures able to predict the clinical behavior of melanoma (e.g., likely sites of metastatic spread) and patient outcome. Most antibody microarrays detect soluble proteins. By contrast, DotScan™ (Medsaic Pty. Ltd., Sydney, Australia) captures live cells that express the corresponding antigens on their surface. The data obtained correlate well with antigen levels determined by flow cytometry [21–23]. DotScan™ has been applied to a variety of clinical samples, including leukaemia, lymphoma, peripheral blood leukocytes from HIV and heart transplant recipients, and colorectal cancer specimens [23–29]. Antibodies printed on the microarray used in the current study were selected based on their association with melanoma disease progression and/or prognosis. Some of these surface molecules are important targets for anti-melanoma drugs, although their functions have not been fully elucidated.

In the present study, we have used an extended DotScan™ microarray [25] to detect surface antigen profiles of live melanoma cells and leukocytes from fresh surgically excised metastatic melanoma LN tumors (AJCC stage IIIb/c disease) and interrogated the findings with prospectively collected clinical and follow up data from each patient. We identified a number of differentially abundant antigens associated with distant metastasis-free and overall survival (OS). In addition, we have identified antigens associated with a prolonged disease-free interval between initial primary melanoma diagnosis and resection of nodal metastatic disease.

## Experimental procedures

### Melanoma lymph node metastases

Melanoma LN metastases (*n* = 38) were obtained from the Melanoma Institute Australia (MIA) Bio-specimen Bank with written informed patient consent and IRB approval (Sydney South West Area Health Service institutional ethics review committee (RPAH Zone) Protocol Nos., X06-0140, X08-0155/HREC 08/RPAH/262, X11-0023/HREC 11/RPAH/32 and X07-0202/HREC/07/RPAH/30). Fresh tumor samples were examined by a specialist melanoma pathologist (RAS) and carefully macro-dissected from non-tumor tissues avoiding necrotic areas and normal lymph node tissue. A high-percentage melanoma tumor content (>80 %) and low necrosis (<20 %) were verified by examining hematoxylin and eosin-stained sections and immunohistochemistry for melanoma antigens S-100 protein, HMB45 and MelanA/MART-1.

The fresh metastatic melanoma LN tumors were collected prospectively over a 13-month period from February 2009 and subsequent clinical follow-up data spaning 24.3–43.1 months (median 35.4 months) post LN resection was obtained. Nine patients were excluded from the study due to a high level of necrosis and poor cell viability (*n* = 3), a non-melanoma diagnosis following subsequent histopathology (*n* = 3) or evidence of distant metastases at diagnosis of the culprit primary melanoma (*n* = 3; stage IV at initial diagnosis). The remaining 29 cases were analyzed for demographic, primary tumor and metastasis pathologic and survival data as detailed in Table 1. As of May 2013, 13 patients were alive [11 with no sign of recurrence (NSR) and 2 with melanoma], 15 patients had died of melanoma and 1 patient had died of unrelated causes. One patient had synchronous AJCC stage IV disease at the time of LN recurrence and was excluded from the survival analyses. Patients with occult primary disease or with previous LN involvement were excluded from the DFI correlation analysis. Despite good cell viability, two patient samples had poor binding of cells to the microarray and were used for Western blot analysis.

**Table 1:** Melanoma patient demographics, pathology, lymph node (LN) recurrence and survival parameters

Demographics				Culprit primary melanoma					Lymph node metastasis				Survival parameters			
No.	Sex	Age	Significant oncology Hx	Sub-type^a^	Site	Bres. thick^b^	Ulc^c^	Mit. rate^d^	AJCC stage^e^	DFI^f^	No. +nodes^g^	LN site	DMF^h^	DMFS^i^	Vital status^j^	OS^k^
1	M	51		NM	R thorax	1.6	y	5	IIIC	8.2	1	R Axilla	y	24.4	NSR	24.4
2	F	46		*unk*	L upper arm	0.6	n		IIIB	16.8	1	L Axilla	y	40.9	NSR	40.9
3	F	35		SSM	L upper arm	1.0	n	3	IIIB	17.9	1	L Axilla	y	38.9	NSR	38.9
4	M	48		SSM	R buttock	1.0	y	0	IIIC	87.1	24	R Ilio-inguinal	y	34.4	A	34.4
5	M	44		*Occult*		*unk*	*unk*	*unk*	IIIC	–	2	R Inguinal	y	35.9	NSR	35.9
6	M	56		SSM	L thorax	1.7	n	5	IIIB	17.5	1	L Axilla	y	35.8	NSR	35.8
7	M	77		SSM	L lumbar	3.3	n	2	IIIC	–	9	R Inguinal	y	22.8	DOC	22.8
8	M	71		NM	R thorax	7.0	y	8	IIIC	–	1	R Axilla	y	35.3	NSR	35.3
9	M	75		NM	L shoulder	6.8	y	4	IIIC	–	2	L Axilla	y	39.5	NSR	39.5
10	F	66		ALM	L sole	1.0	*unk*	*unk*	IIIB	98.2	1	L Inguinal	y	35.5	NSR	35.5
11	M	41		*Occult*		*unk*	*unk*	*unk*	IIIB	–	1	L Axilla	y	35.0	NSR	35.0
12	M	41		SSM	R upper arm	0.7	n	*unk*	IIIB	19.0	1	R Axilla	y	29.7	NSR	29.7
13	M	69		NM	R shoulder	4.1	y	9	IIIC	13.2	1	R Axilla	y	29.8	NSR	29.8
14	M	47	Thyroid papillary carcinoma	SSM	R ankle	0.8	n	2	IIIB	96.6	1	R Sub-inguinal	n	17.0	A	43.2
15	M	58		SSM	L forearm	1.6		2	IIIC	92.6	1	L Axilla	n	29.7	D	36.3
16	F	88	Pituitary adenoma	NM	R shin	3.5	n	8	IIIC	30.1	4	R Inguinal	n	17.8	D	22.2
17	M	83		NM	L forearm	2.2	y	5	IIIC	3.6	1	L Axilla	n	15.6	D	22.3
18	M	52	Metastatic carcinoma unknwn primary	NM	L palm	5.5	y	11	IIIC	–	1	L Axilla	n	1.8	D	12.3
19	M	47		SSM	L forearm	0.6	n	3	IIIC	247.2	69	L Axilla & L Neck	n	12.7	D	20.8
20	F	38	Thyroid papillary carcinoma	NM	L upper arm	3.5	y	17	IV	10.3	2	R Inguinal	n		D	31.7
21	F	80		SSM	L calf	2.2	n	2	IIIC	36.6	4	L Inguinal	n	4.4	D	10.2
22	M	78		NM	R thorax	2.0	n	0	IIIC	25.4	18	R Axilla	n	25.6	D	27.6
23	M	70		SSM	R shin	2.3	y	4	IIIC	7.3	9	R Ilio-inguinal	n	1.7	D	4.0
24	M	83		NM	L calf	3.6	n	35	IIIC	18.8	8	L Ilio-inguinal	n	10.3	D	12.8
25	M	68		*Nk*	R calf	0.6	*unk*	0	IIIC	19.1	2	R Inguinal	n	16.9	D	29.3
26	F	60		DM	L calf	1.2	y	2	IIIC	27.4	1	L Ilio-inguinal	n	3.1	D	15.7
27	M	61		DM	R shoulder	4.5	n	12	IIIC	23.2	7	R Axilla	n	22.7	D	22.7
28	M	71		NM	R neck	4.0	n	2	IIIC	9.2	2	R Neck	n	10.4	NSR	10.4
29	F	89		NM	R calf	3.3	y	6	IIIC	26.7	8	R Inguinal	n	21.6	D	21.6

^a^Primary melanoma sub-type: *NM* nodular melanoma, *Nk* not known; *SSM* superficial spreading melanoma, *Occult* no primary lesion detected, *DNM* desmoplastic neurotropic melanoma

^b^Breslow thickness in mm

^c^Ulceration, *y* present, *n* absent

^d^Mitotic rate per mm^2^

^e^American Joint Committee on Cancer (AJCC) staging at time of lymph node recurrence

^f^ *DFI* Disease-free interval, time between diagnosis of culprit primary lesion and lymph node resection (months)

^g^Number of positive lymph nodes containing metastatic melanoma

^h^ *DMF* Distant metastasis-free parameter, *y* distant metastasis free, *n* recorded distant metastasis/es

^i^ *DMFS* Distant metastasis-free survival, time between lymph node resection and appearance of distant metastasis or last clinical follow-up (months)

^j^Vital status at end of reporting period, May 2013, *NSR* no sign of recurrent disease, *A* Alive with melanoma; *D* Melanoma-related death, *DOC* Died from other causes unrelated to melanoma

^k^ *OS* Overall survival, calculated from the time of LN resection to death or the last clinical follow-up (months)

In summary, we investigated the associations of CD45^−^ enriched melanoma cell (*n* = 25) and CD45^+^ leukocyte (*n* = 23) immuno-profiles in melanoma patients with distant metastasis-free survival (DMFS) and OS post LN resection of stage IIIb/c disease. Immuno-profiles of CD45^−^ enriched melanoma cells (*n* = 20) and CD45^+^ leukocytes (*n* = 19) were also correlated with DFI (time between diagnosis of culprit primary melanoma and LN resection; see Supplementary Table 1).

### Preparation of viable, single cell suspensions

Fresh tumor samples were placed in Hanks’ balanced salt solution (HBSS; Sigma-Aldrich, St. Louis. MO, USA) at 4 °C and processed within 16 h to ensure cell viability. Samples were finely diced with a scalpel blade and incubated in a dissociation buffer containing 1 % (w/v) collagenase type IV (Worthington, Lakewood, NJ, USA) and 0.2 % (w/v) DNase I (bovine pancreas; Sigma-Aldrich) in HBSS at 37 °C for 1 h, with gentle vortexing every 15 min. The semi-digested tissue was then passed through a fine wire mesh strainer and the resulting suspension centrifuged (450 × *g,* 4 °C, 5 min). Small aggregates were removed using 200 μm and then 50 μm Filcon cup filters (BD Biosciences, San Jose, CA, USA), resulting in a single-cell suspension with 70–90 % viability, that was frozen at −80 °C in fetal calf serum (FCS) with 10 % DMSO until required.

### Separation of leukocytes from melanoma cells

Prior to surface profiling, samples were thawed and pelleted (400×*g*, 4 °C, 5 min) and the cells resuspended in RPMI growth medium with 0.2 % (w/v) DNase I for 10 min at room temperature. Leukocytes were bound to CD45 antibody microbeads (Miltenyi Biotec, Auburn, CA, USA) and separated from cell suspensions using an AutoMACS™ Pro Separator (Miltenyi Biotec) as per the manufacturer’s instructions. Briefly, 2 × 10^7^ cells were incubated with 40 μL anti-CD45 microbeads in a buffer containing PBS with 0.1 % BSA, 2 mM EDTA and 2 % AB serum at 4 °C for 10 min. Cells were diluted, pelleted (300×*g*, 4 °C, 10 min), resuspended in 500 μL of the above buffer and passed through a 50 μm filter before removal of magnetic beads with attached CD45^+^ cells using the ‘deplete’ setting on the AutoMACS™ Pro Separator. To confirm depletion of the CD45^+^ cells, a melanoma LN cell suspension was labelled with mouse monoclonal anti-CD45-PE or anti-IgG2a-PE (Miltenyi Biotec) and analyzed on a FACScalibur™ (BD Biosciences) before and after immuno-magnetic separation (Supplementary Fig 1).

### Capture of cells on DotScan™ microarrays

The microarrays were constructed as duplicate dots (10 nl) on nitrocellulose-coated slides (Grace Bio-labs Inc., Bend, OR, USA) as previously described [25]. All array antibodies were specific to extracellular domain sequences where possible, if not the full-length protein. However, antibody avidity might be reduced following its immobilization to the nitrocellulose slide. CD45^+^ and CD45^−^ live cell suspensions were suspended at a density of 1.3 × 10^7^/ml in growth medium (RPMI with 10 % FCS and 2 % heat-inactivated human AB serum) and incubated on pre-moistened microarrays in a humidified chamber at 37 °C for 30 min. Captured cells were fixed in 3.7 % formaldehyde for 30 min at room temperature and washed in PBS. Arrays were imaged directly with an optical scanner (Medsaic Pty Ltd) without staining or labelling, and analyzed using DotScan™ software (Medsaic Pty Ltd) [23]. This software quantifies the density of cell binding on each antibody dot above background levels, on an 8-bit scale from 1–256. Dot intensities on an array reflect the proportion of cells expressing each antigen and/or the level of expression of a particular antigen per cell. The average numbers of cells bound to each dot, determined microscopically, correlate well with average binding density values. The number of cells captured on an antibody dot in the microarray also depends on the affinity of the antibody-antigen interaction.

### Statistical analysis of antibody microarray results

Cell binding densities were corrected for background and isotype-control binding and the duplicate array information was averaged and log_2_-transformed. Microarray data, consisting of 52 antigens for melanoma (CD45^−^) samples and 78 antigens for leukocytes (CD45^+^), were median normalized separately. DMFS was calculated from the time of LN resection to appearance of distant metastasis or last clinical follow-up. OS was calculated from the time of LN resection, to last clinical follow-up or death. Univariate (log-rank test) and multivariate (Wald’s test) survival models were used to assess associations between antigen levels and patient survival (DMFS and OS). Due to the small patient numbers (enriched melanoma cells, *n* = 25 and leukocytes, *n* = 23), we were unable to include all clinical variables in a single survival model. Instead, we restricted our attention to the patient’s age, gender and AJCC stage at LN resection (stage IIIb or IIIc) because these are the strongest known predictors of outcome in AJCC stage III melanoma patients. For patients with multiple primary melanomas (*n* = 3), the culprit primary was designated on the basis of the presence of the most adverse prognostic factors and its anatomic site, due to a greater likelihood that it was the source of the metastatic disease, as previously described [30, 31]. We censored patients who died of other causes, and for the DMFS analysis, patients who had no distant metastasis. For the DFI correlation analysis, Pearson’s r^2^ values were calculated to determine the relationship between surface antigen levels and the time (in months) between the culprit primary melanoma diagnosis and LN resection of stage III metastatic disease.

### Western blotting

Total protein extracts were made from CD45^+^ cell populations from 13 LN samples (see Supplementary Table 1) by lysing cells in a denaturing urea buffer containing 7 M urea, 2 M thiourea, 40 mM Tris, 65 mM DTT supplemented with 0.1 mM phenylmethylsulfonyl fluoride (PMSF) and centrifuged (16,000×*g*, 15 min, 4 °C). Due to limited sample availability, the level of one surface antigen (CD55) was confirmed by Western blotting. Proteins were separated by 10 % SDS-PAGE and transferred to a PVDF membrane at 400 mA for 1 h using a Criterion™ Blotter (BioRad, Hercules, CA, USA). The membrane was blocked in 5 % (w/v) skim milk and incubated overnight at 4 °C with a 1:5,000 dilution of rabbit monoclonal antibody against CD55 (EPR6689, Epitomics, Inc., Burlingame, CA, USA) and 1:10,000 dilution of mouse monoclonal antibody against succinate dehydrogenase subunit A (SDHA; Santa Cruz Biotechnology, Inc., Santa Cruz, CA, USA). Membranes were then incubated with donkey, anti-rabbit (Abcam, Waterloo, NSW, Australia) and goat, anti-mouse (Santa Cruz) secondary antibodies conjugated to horseradish peroxidase for 2 h at room temperature. Bands were visualized with ECL detection reagent and imaged using chemiluminescence film (GE Healthcare, Piscataway, NJ, USA). Bands were quantified using ImageQuantTL density analysis software (GE Healthcare). An average of the density of SDHA and the total protein stain was used as an internal loading control. To confirm the association with DMFS, CD55 level ratios were tested using the log-rank test described above.

## Results

### Surface profiles of melanoma LN metastases

**Fig. 1:**
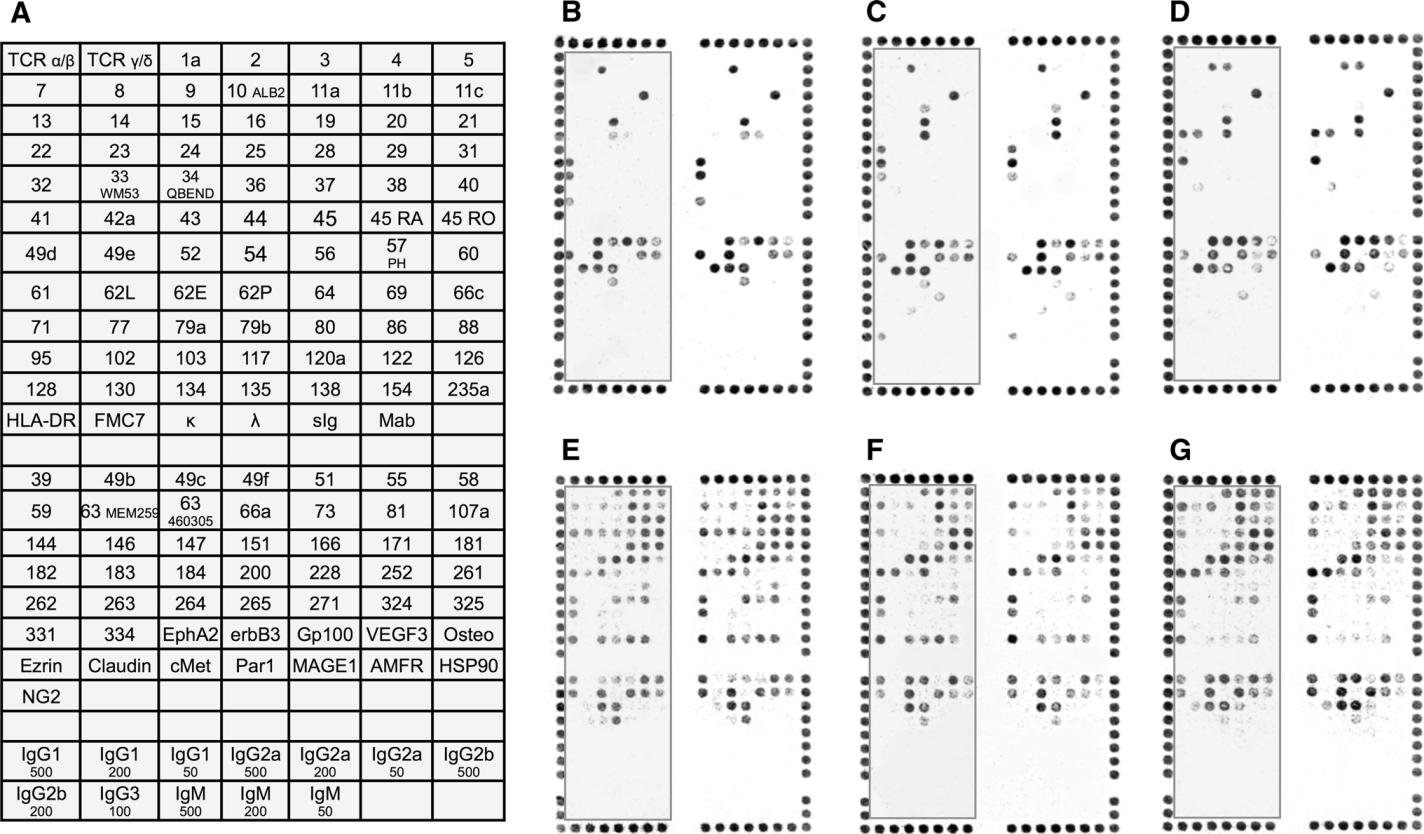
Immuno-phenotypes of cell suspensions isolated from lymph node tumors resected from stage III metastatic melanoma patients. **a** Antibody key for the microarray, printed in duplicate on nitrocellulose slides. Antibody key is zone framed in grey on (**b**–**g**). (**b**–**d**) Antibody binding patterns of enriched melanoma cells (CD45^−^) from patients 19, 13 and 8 respectively. (**e**–**g**) Antibody binding patterns of leukocytes attached to CD45^+^ magnetic beads (CD45^+^) isolated from patients 19, 25 and 7 respectively. The *numbers* in (**a**) refer to antibodies against corresponding CD antigens; IgG1, IgG2a, IgG2b, IgG3 and IgM are murine isotype control antibodies tested at the concentrations shown in μg/mL; TCR α/β, TCR γ/δ, HLA-DR, FMC7, k and λ are antibodies against T-cell receptors α/β and γ/δ, HLA-DR, FMC-7, kappa and lambda immunoglobulin light chains, respectively. A border of CD44/CD29 *alignment dots* is shown around the duplicate microarrays of (**b**–**g**)

Single cell suspensions of 29 metastatic melanoma LN specimens were immuno-magnetically separated to isolate CD45^+^ leukocytes and enrich for melanoma cells (CD45^−^). Cell separation was confirmed by flow cytometry (Supplementary Fig. 1). This together with a failure of the CD45^−^ cells to bind CD45, CD45RA and CD45RO or other leukocyte specific antigens on the arrays confirmed the effectiveness of the cell separation. Adequate cell numbers (4 × 10^6^ cells), viability (>70 %) and binding were achieved for CD45^−^ and CD45^+^ cell populations from 26 to 24 patient samples, respectively (See Fig. 1 for optical scan of cell binding patterns). Cell binding to 52 and 78 antibodies was observed for CD45^−^ enriched melanoma cells and CD45^+^ leukocytes, respectively. Normalization and subsequent analyses were performed using this these data. Some antigens were expressed at variable levels on all CD45^−^ fractions, e.g., CD29, CD44, CD51, CD63, CD71, CD146 and CD151. Although the leukocyte (CD45^+^) antigen profiles were more diverse, perhaps reflecting the heterogeneity of the cell populations, as expected, there was consistent binding to the antibodies for CD45, and CD45RA and/or CD45RO for all patient samples.

### DMFS and OS analyses

**Fig. 2:**
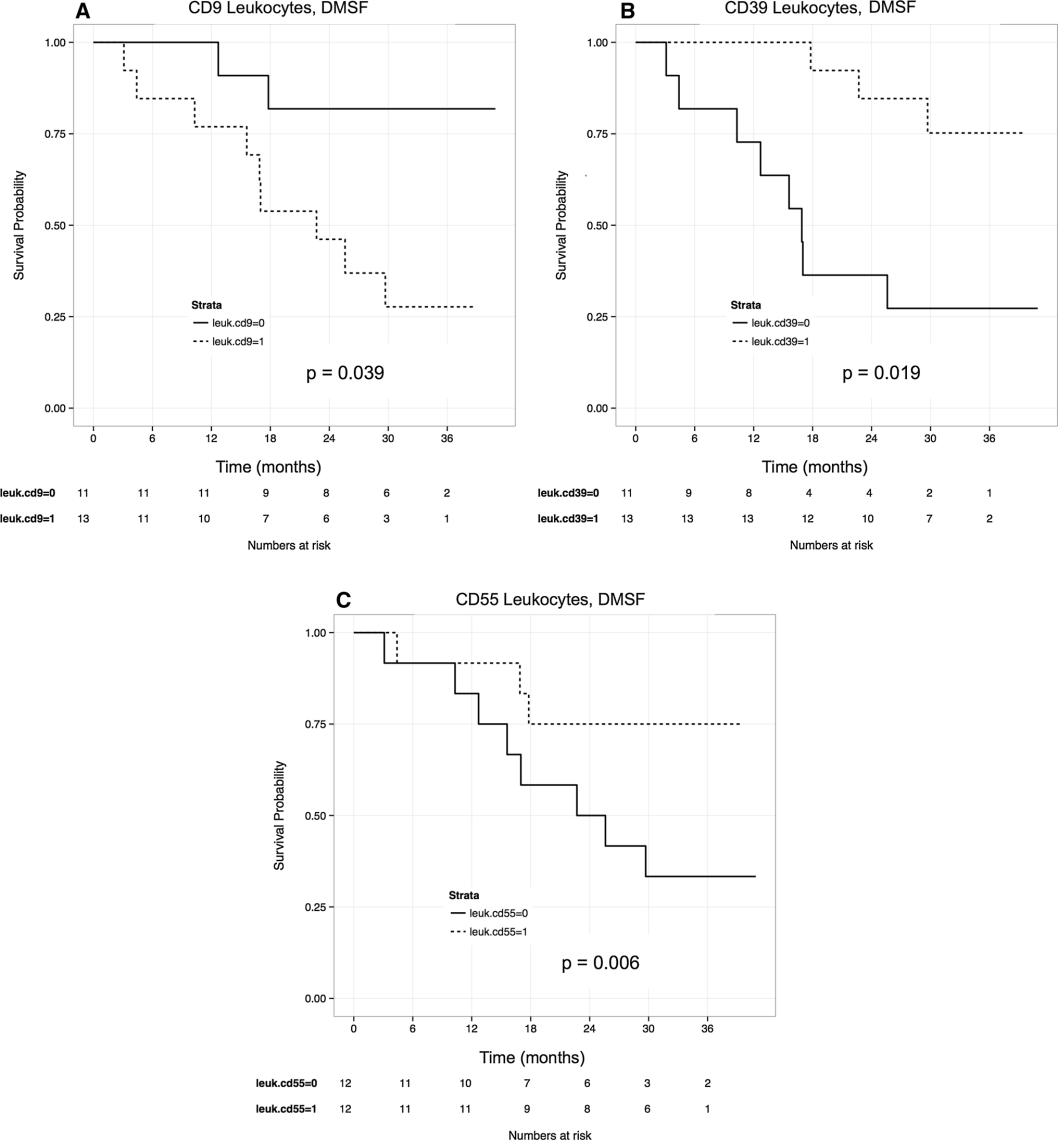
Univariate cox proportional hazard models were used to evaluate associations between surface antigen levels and distant metastasis-free survival (DMFS). For graphical representation, plotted variables were dichotomized based on median antigen levels, 0 = low, 1 = high. Increased levels of (**a**) CD9 on CD45^+^ leukocytes were significantly associated with with distant metastasis and poor survival outcomes. Conversely, higher levels of (**b**) CD39 and (**c**) CD55 on CD45^+^ leukocytes were associated with DMFS

In leukocyte (CD45^+^) fractions, high levels of CD9 were associated with decreased DMFS on both univariate and multivariate analyses (HR = 2.67, *p* = 0.039 and HR = 3.73, *p* = 0.036, respectively; Fig. 2). In contrast, increased levels of CD39 and CD55 were associated with increased DMFS on univariate (HR = 0.29 and 0.28, *p* = 0.019 and 0.006, respectively) and multivariate analyses (HR = 0.01 and 0.10, *p* = 0.004 and 0.005, respectively). A significant association between increased levels of CD55 and DMFS was confirmed by Western blotting (HR = 2.8; *p* = 0.008; see Fig. 4). The CD45^−^ enriched melanoma cells displayed differential binding to CD117 (c-KIT), with higher levels associated with decreased DMFS on univariate analysis with borderline significance (HR = 1.56; *p* = 0.07).

**Fig. 3:**
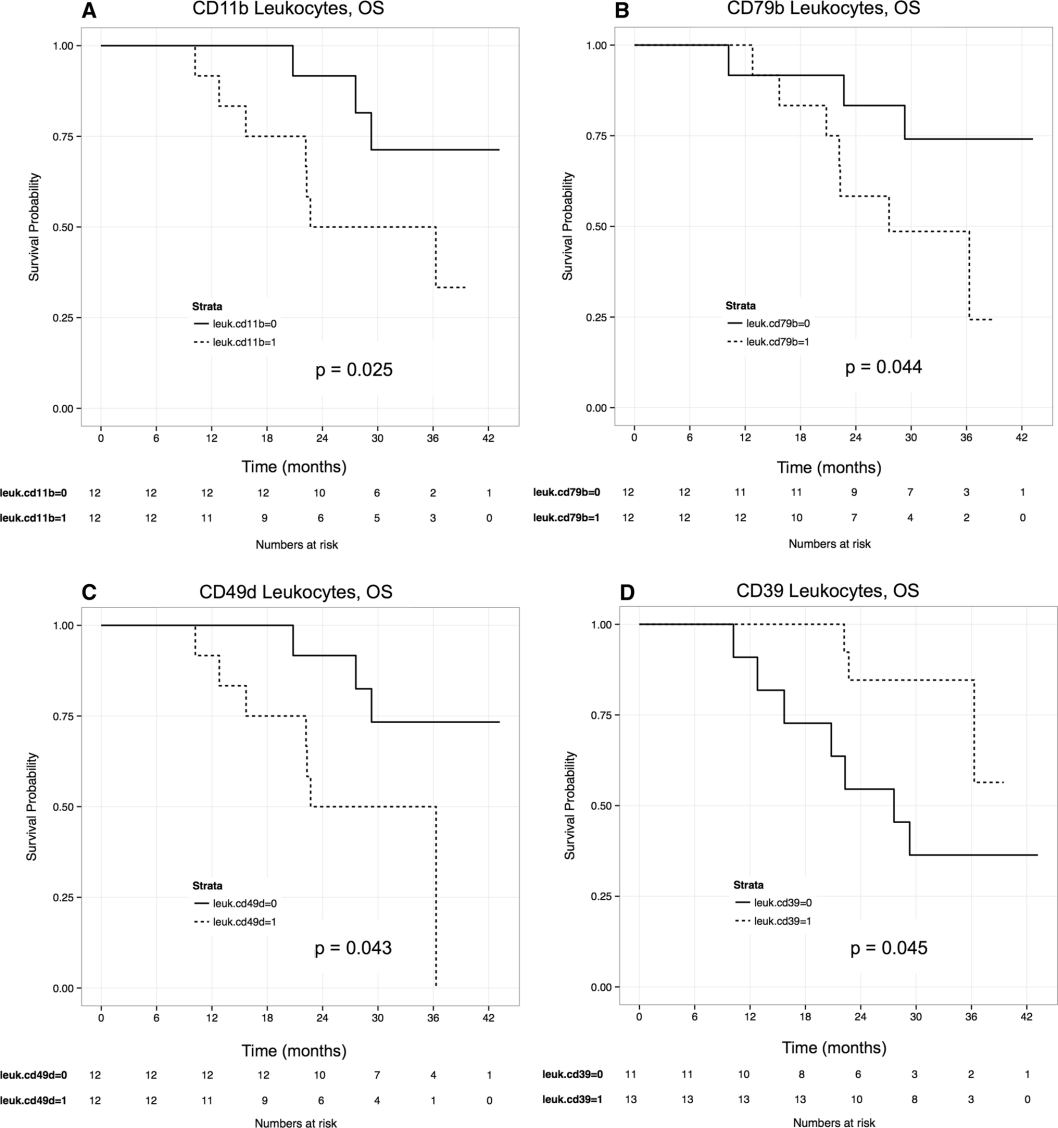
Univariate cox proportional hazard models were used to evaluate associations between surface antigen levels and overall survival (OS). For graphical representation, plotted variables were dichotomized based on median antigen levels, 0 = low, 1 = high. Increased levels of (**a**) CD11b, (**b**) CD79b and (**c**) CD49d on CD45^+^ leukocytes were significantly associated with reduced overall survival, whereas higher levels of (**d**) CD39 was associated with increased survival

Higher levels of CD39 on CD45^+^ leukocytes were also significantly associated with increased OS using both univariate and multivariate models (HR = 0.29, *p* = 0.045 and HR = 0.10, *p* = 0.016, respectively; Fig. 3). There is a documented synergy between CD39 and CD73 activities [23]. We found increased CD73 levels associated with DMFS and OS, with marginal significance (*p* = 0.08 and 0.13, respectively). When CD73 and CD39 levels were modelled together, CD39 remained significantly associated with DMFS (*p* = 0.027). Increased levels of CD11b, CD49d and CD79b on CD45^+^ leukocytes were associated with reduced OS (HR = 2.72, 2.46, 1.76, *p* = 0.025, 0.043, 0.044, respectively). However these associations were not statistically significant on multivariate analysis. Leukocyte results are summarised in Table 2 and survival plots for DMFS and OS are depicted in Figs. 2 and 3. No statistically significant association with OS was found for the 58 antigens tested on CD45^−^ enriched melanoma cells.

**Table 2:** Leukocyte antigens significantly associated with survival

Acc. #^a^	Antigen/protein name	Univariate		Multivariate^d^	
		HR^b^	*p* value^c^	*HR* ^b^	*p* value^e^
Distant metastasis-free survival					
P12926	CD9 (tetraspanin-29)	2.67	0.039	3.73	0.036
P49961	CD39 (ecto-ATPase diphosphohydrolase 1)	0.29	0.019	0.010	0.004
P08174	CD55 (complement decay-accelerating factor)	0.28	0.006	0.098	0.005
Overall survival					
P11215	CD11b (integrin αM)	2.72	0.025	1.75	>0.2
P49961	CD39 (ecto-ATPase diphosphohydrolase 1)	0.29	0.045	0.099	0.016
P13612	CD49d (integrin α4)	2.46	0.043	1.22	>0.2
P40259	CD79b (B-cell antigen receptor complex-associated protein β chain)	1.76	0.044	1.36	>0.2

^a^Accession numbers were obtained from UniProtKB/Swiss-Prot knowledgebase (www.uniprot.org)

^*b*^ *HR* Hazard ratio

^c^Log-rank test, significance level *p* < 0.05

^d^Multivariate survival model, including age, gender and AJCC stage at lymph node recurrence

^e^Wald test, significance level *p* < 0.05

### DFI correlation analyses

A proportion of patients with primary melanoma carry a risk for recurrence and distant metastases after a symptom-free period that can span decades, despite favourable prognostic determinants from their primary tumor. The relationship between this DFI, defined as the time between primary melanoma resection and resection of nodal metastases (spanning 3.6–247.2 months in this study) and antigen levels was also investigated. Enriched melanoma (CD45^−^) cells showed higher levels of 11 surface antigens in resected LNs from metastatic melanoma patients with a prolonged DFI (*p* < 0.05; average r^2^ of 0.484; see Supplementary Fig. 2a and Table 3). Five of these antigens are cell adhesion molecules, i.e., integrins (CD29, CD49c), tetraspanin CD63, CD56 (neural adhesion molecule, NCAM) and CD107a (lysosome-associated membrane protein 1). For CD45^+^ leukocyte fractions, nine antigens were correlated with shorter DFI (*p* < 0.05; average r^2^ of −0.590; Supplementary Fig. 2b and Table 3). Of these, eight antigens are related to T cell immunity, i.e., CD7, CD8, CD43, CD54, CD56, CD103, CD134 and CD166.

**Table 3:** Antigens with significant correlation to the disease-free interval

Acc. #^a^	Antigen/protein name	r^2^ ^b^	*p* value^c^
CD45 enriched melanoma cells			
P05556	CD29 (Integrin β1)	0.494	0.0267
P28906	CD34 (Hematopoietic progenitor cell antigen CD34)	0.489	0.0295
P28907	CD38 (ADP-ribosyl cyclase 1)	0.451	0.0460
P17301	CD49c (Integrin α3)	0.462	0.0400
P13591	CD56 (Neural cell adhesion molecule 1; NCAM1)	0.452	0.0454
P13987	CD59 (Membrane attack complex inhibition factor)	0.485	0.0301
P02786	CD71 (Transferrin receptor protein 1)	0.462	0.0400
P04216	CD95 (Apoptosis-mediating surface antigen FAS)	0.593	0.0058
P08962	CD63 (Tetraspanin-30; Lysosomal-associated membrane protein 3; Melanoma-associated antigen ME491)	0.475	0.0344
P11279	CD107a (Lysosome associated membrane protein 1; LAMP-1)	0.489	0.0286
P41217	CD200 (OX-2 membrane glycoprotein)	0.472	0.0358
CD45^+^ Leukocytes			
P09564	CD 7 (T-cell antigen CD7)	−0.542	0.0164
	CD 8 (T-cell glycoprotein CD8) – α (Acc. # P01732) and β (Acc. # P10966) chains	−0.677	0.0014
P16150	CD 43 (Leukosialin)	−0.481	0.0370
P05362	CD 54 (Intercellular adhesion molecule 1; ICAM1)	−0.642	0.0030
P13591	CD 56 (Neural cell adhesion molecule 1; NCAM1)	−0.522	0.0219
P11911	CD 79a (Surface IgM-associated protein)	−0.523	0.0217
P38570	CD 103 (Integrin E)	−0.550	0.0146
P43489	CD 134 (Tumor necrosis factor receptor superfamily member 4)	−0.614	0.0052
Q13740	CD 166 (Activated leucocyte cell adhesion molecule)	−0.758	0.0002

^a^Accession numbers were obtained from UniProtKB/Swiss-Prot knowledgebase (www.uniprot.org)

^b^Pearson product momentum coefficient, r^2^. r^2^ > 0 is indicative of a positive relationship between antigen expression levels and a long disease-free interval, whereas r^2^ < 0 describes decreased antigen levels associated with a short disease-free interval

^c^Unadjusted *p* values, significance level *p* < 0.05

## Discussion

Our novel methodology combines immuno-magnetic cell separation with surface antigen profiling (partial membrane proteome) to profile separated leukocyte (CD45^+^) and enriched melanoma cell (CD45^−^) populations. Metastatic melanoma is highly immunogenic and by profiling leukocytes separately, we can understand how melanoma cells might escape or diminish immune system control within LNs and progress to metastatic disease. Some antigens showed limited binding or were undetected across all CD45^+^ or CD45− cell populations and were removed from the analyses. Low cell capture may be due to low antigen levels, inaccessible antigen epitopes or a reduced affinity of antibodies following immobilization on nitrocellulose. Surface antigens significantly associated with DMFS, OS and DFI are discussed below.

### Survival analyses

#### Leukocyte antigens associated with reduced survival

Patients with shorter OS showed enhanced levels of CD79b, part of the B-cell receptor complex (BCR), implicating an elevation of B-lymphocytes in LNs resected from stage III metastatic melanoma patients with poor survival outcomes. De novo lymphatic formation or lymphangiogenesis is associated with cancer metastasis [32–34]. B-lymphocyte accumulation within draining LNs is required for LN lymphatic sinus expansion in response to tumor growth and B-cell-associated lymphangiogenesis is required to increase lymph flow through the tumor-draining LNs [35]. These changes could actively promote tumor dissemination through the lymphatic system [36]. In murine models of squamous cell carcinoma, wild-type mice showed tumor draining LN B-cell accumulation and lymphangiogenesis and the extent of these alterations predicted progression from benign papillomas to metastatic carcinomas [37]. Primary melanomas prepare tumor-draining LNs for the seeding of metastatic disease by stimulating lymphangiogenesis and dampening sentinel node immunity prior to the arrival of malignant cells [35, 38]. A multivariate risk analysis revealed that tumor lymphangiogenesis was the most sensitive prognostic marker for melanoma sentinel LN metastasis [39]. Here, an accumulation of B-lymphocytes, indicated by elevated CD79b, may be related to lymphangiogenesis, increased lymph flow and subsequent tumor dissemination in stage III metastatic melanoma patients with reduced OS. Interestingly, protective cell-mediated immunity provoked by adenoviral-based cancer vaccines is enhanced in the absence of B-cells, suggesting that a therapeutic regimen that includes depletion of B-lymphocytes may be beneficial [40].

We detected higher levels of integrins α_M_ (CD11b) and α_4_ (CD49d) on CD45^+^ leukocytes in LN tumors from patients with reduced OS. CD11b is found primarily on innate immune cells of myeloid lineage, and plays important roles in cell adhesion, migration, chemotaxis, and phagocytosis [41]. Melanoma cell extravasation was decreased following a reduction in integrin α_M_β_2_ levels on polymorphonuclear neutrophils by functional blocking of IL-8 binding [42], implicating CD11b in melanoma metastasis. Myeloid-derived suppressor cells (MDSCs) are over-produced in tumor-bearing hosts, constituting approximately 5 % of total cells in tumors [43]. MDSCs are CD11b^+^/Gr-1^+^ (marker for granulocytes) and contribute significantly to immune escape, intravasation and angiogenesis [44]. These potent immunosuppressors can elicit systemic effects on secondary lymphoid organs as well as local effects within the tumor microenvironment [44] and can compromise the efficacy of cancer immunotherapy [45, 46]. CD11b^+^ MDSCs were enriched in melanomas and lymphatic organs during tumor progression [47]. Recently, two sub-populations of MDSCs were described, based on the expression of CD49d. The CD49d^+^ subset of MDSCs is mainly monocytic and suppresses antigen-specific T cell proliferation in a nitric oxide-dependent mechanism, more potently than CD11b^+^/CD49d^−^ MDSCs [48]. The association between reduced OS and increased levels of CD11b^+^ and CD49d^+^ on leukocytes from LNs containing metastatic melanoma may be attributed to MDSCs capable of potentiating local immunosuppression.

Levels of CD9 were increased on CD45^+^ leukocytes isolated from LNs resected from melanoma patients with reduced DMFS. CD9 is a tetraspanin, expressed on immune cells of lymphoid and myeloid lineages. By treating peripheral natural killer (NK) cells with transforming growth factor (TGF)-β1 under hypoxic culture conditions, Cedeira et al. generated a population of CD9^+^ NK cells that secreted pro-angiogenic factors and have reduced cytotoxicity [49]. TGF-β1 serum levels positively correlate with melanoma stage and higher levels are prognostic for reduced OS [50]. NK cells that express CD9 were thought to only occur in the female reproductive system, however a CD9^+^ NK cell population was recently detected in the peripheral blood of metastatic melanoma patients [51]. Altered NK cell function is a component of global immune dysregulation that occurs in advanced malignancies and a number of NK cell-based immunotherapies of melanoma are under investigation. Further analysis of the role of CD9^+^ leukocytes in progression of metastatic melanoma is warranted.

#### Leukocyte antigens associated with increased survival

**Fig. 4:**
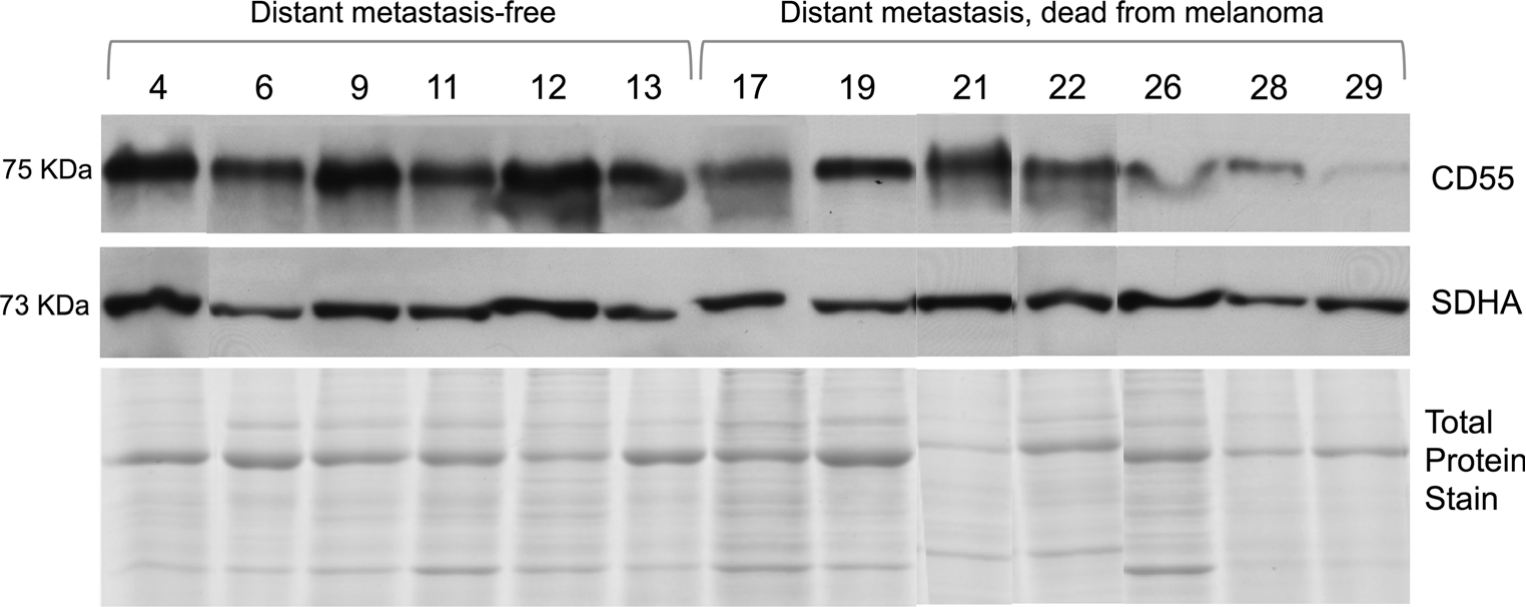
Western blot verifying increased CD55 on CD45^+^ leukocytes fractionated from Stage III metastatic melanoma patients with distant metastasis-free survival (HR = 2.8; *p* = 0.008). Succinate dehydrogenase subunit A (SDHA) and total protein stains were used as loading controls

Higher CD55 (decay-accelerating factor) levels on leukocytes were associated with longer DMFS on univariate and multivariate analyses (*p* = 0.006 and 0.005), a finding confirmed by Western blot analysis (Fig. 4; *p* = 0.008). CD55 is a ubiquitous complement regulatory protein that is critical for protection from bystander complement-mediated attack. Enhanced levels of CD55 may simply protect leukocytes from complement-mediated lysis. Interestingly, in our recent comprehensive proteomic analyses of lymph nodes resected from stage IIIc melanoma patients, several complement proteins (complement C3, clustin and complement component 1Q sub-component-binding protein) were differentially abundant between patients with good (>4 years survival) and poor (<1 year survival) prognoses (*n* = 33). Moreover, the top ranking (FDR < 0.01) pathway maps generated by MetaCore™ (GeneGO™, Encinitas, CA, USA) involved complement pathways (S. Mactier et al. unpublished data, 2013). CD55 also co-stimulates T-cells when engaged and cross-linked by antibodies [52, 53]. CD55 is also a ligand for CD97 and may be required to stabilize T-cell receptor-MHC engagement to enhance T-cell function [54]. Although this finding was confirmed using Western blot (*n* = 13; 2 patients independent of microarray analysis), investigation of CD55 antigen levels on leukocytes in a larger patient cohort is required.

High levels of CD39 (Ecto ATP-diphosphohydrolase 1) on CD45^+^ leukocytes were associated with increased OS and DMFS. CD39 is expressed by a broad range of immune cells and catalyzes the sequential hydrolysis of ATP to AMP that is further degraded to anti-inflammatory adenosine. In the immune system, extracellular ATP functions as a “natural adjuvant” that exhibits multiple pro-inflammatory effects and is known to boost immune responses in the tumor microenvironment. Deletion of CD39 in a murine model abrogated angiogenesis, leading to reduced growth and metastasis of subcutaneous melanoma tumors [55]. However, our results show that the presence of higher CD39 on CD45^+^ leukocytes confers a survival advantage. In the context of LN metastases, reduced CD39 levels would allow extracellular ATP to trigger a more pronounced inflammatory response [56] that can induce lymphangiogenesis and promote tumor cell dissemination [36]. Therefore, opposed to the documented immunosuppressive properties, higher CD39 levels in patients with longer DMFS and OS may reflect reduced lymphatic dissemination of metastatic melanoma. As CD73 works in concert with CD39 to catalyze the conversion of AMP to adenosine [57], we examined levels of CD73 in our dataset. Increased CD73 on CD45^+^ leukocytes were associated with DMFS with marginal significance. Although CD39 and CD73 require further investigation in a larger patient cohort, we have observed a trend of increased co-expression levels on CD45^+^ leukocytes from patients with longer DMFS.

#### CD117 is associated with distant metastasis and poor survival on enriched melanoma cells

Increased levels of the receptor tyrosine kinase CD117 (c-KIT) on enriched melanoma cells (CD45^−^) were associated with distant metastasis and poor survival outcomes but this was not statistically significant (*p* = 0.07). The reversible expression of surface antigens on melanoma cells [58] as well as the presence of other non-hematopoietic cell types could explain the limited stratification of the CD45^−^ cell population obtained in our study.

### Surface antigens associated with a prolonged disease free interval

The time interval between removal of the primary melanoma and detection of metastatic disease is very variable and can span many years, or even decades. The mechanisms by which metastatic disease may be present but remain clinically undetected for such prolonged periods, presumably influenced by interactions between the melanoma phenotype and the patient’s immune system, have not received sufficient attention to date [59]. A better understanding of the molecular cross-talk between tumor cells, immune cells and the extracellular matrix in secondary sites, and how these regulate metastatic disease progression, may lead to improved therapeutic strategies to eradicate them or at least to induce or maintain disseminated tumor cells in a clinically-silent chronic disease (“dormant”) state [60]. Adjuvant therapies, such as antibodies that target metastatic melanoma cells are of considerable interest [61, 62]. We performed correlation analyses of antigen levels and the DFI (time from primary diagnosis and detection of LN metastasis) to identify surface antigen signatures associated with melanoma progression following prolonged disease-free (latent) intervals.

#### Cell adhesion markers on enriched melanoma cells (CD45^−^) correlate with longer disease-free intervals

The tumor microenvironment is a critical regulator of cancer progression [60] and integrin-mediated interactions between tumor cells and the extracellular matrix (ECM) modulate the metastatic potential of tumor cells [63]. We identified higher levels of the integrins CD29 and CD49c on CD45^−^ cells from metastatic LN tumors following a prolonged DFI. Integrins are trans-membrane proteins involved in cell adhesion, migration, proliferation and signal transduction [64]. To escape a dormant state, tumor cells must engage with the ECM via integrin receptor(s), inducing signalling that leads to cytoskeletal reorganisation and cell proliferation [60]. Integrin-containing complexes interact with the ECM via actin filaments and focal adhesion complexes, but also regulate, and are regulated by receptor tyrosine kinases and other signalling pathways [65, 66]. There is growing evidence implicating CD29 (integrin β1) heterodimers and signal transducers in regulating tumor cell dormancy [63, 67–69]. Other antigens involved in cell adhesion were increased on CD45^−^ cells following reactivated dormant disease, including CD56, CD63 and CD107a.

#### Reduced levels of T-cell activation markers in LN metastases resected after a prolonged disease-free interval

Metastatic melanoma is highly immunogenic, however attempts to boost patient immunity and/or vaccinate against melanoma have had limited success [70, 71]. Melanoma cells are capable of immune subversion within a tumor or draining LN by altering dendritic cell function through the secretion of tumor-derived cytokines [72], leading to the generation of suppressive and regulatory T-cells [73]. T-cell-mediated immunity is an important component in the regulation of tumor dormancy [74]. In transgenic mouse models, CD8^+^ T-cells appear to halt the expansion of disseminated melanoma cells in the bone marrow and LNs [75]. Moreover, CD8^+^ T-cells appear to have distinct roles in controlling disease progression and metastatic spread [76], suggesting that the effect of immune cells on melanoma dormancy might depend on the microenvironment [59]. Here we found a significant correlation between reduced CD8 T-cells in patients with stage III metastatic disease resected following a long DFI. Interestingly, 7 other antigens involved in T-cell immunity were reduced in these patients, including CD7, CD43, CD54 [77], CD56 [78], CD103 [79], CD134 [17] and CD166 [80]. Decreased CD134 levels on CD4^+^ T-cells in sentinel LNs draining primary melanomas correlated with more advanced tumor features and nodal involvement [17]. Overall, these results suggest that a local immuno-suppressive LN microenvironment may induce the reactivation and expansion of dormant tumor cells.

## Conclusions

Surface markers prognostic for survival identified here require validation in a larger, independent cohort of stage III melanoma patients. The significant findings of low CD9 (DMFS) and high CD55 (DMFS) and CD39 (DMFS and OS) levels on CD45^+^ leukocytes with better survival outcomes on multivariate analysis warrants further study. Enhanced expression of the adhesion markers CD29, CD49c, CD56, CD63 and CD107a on CD45^−^ melanoma cells and loss of T-cell-related immunity is implicated in melanoma progression following a prolonged period in which metastatic disease has remained clinically silent/undetected. A better understanding of the interplay of melanoma cells, the immune system and the factors that control them will enable more accurate prognostication and furthermore, provide new therapeutic approaches to treat this complex, heterogeneous cancer.

## Electronic supplementary material

Below is the link to the electronic supplementary material.

To confirm the effective immuno-magnetic depletion of CD45^+^ leukocytes, a melanoma LN cell suspension was labelled with anti-CD45-PE or anti-IgG2a-PE and analyzed by flow cytometry. Overlays of CD45-PE and IgG2a-PE labelled cells (**a**) before and (**b**) after CD45-conjugated microbead depletion, achieved an ~ 100-fold reduction of CD45 + leukocytes

Scatter plots depicting significant correlations between disease-free interval (months) and surface antigen levels on CD45^−^ enriched melanoma cells

Scatter plots depicting significant correlations between disease-free interval (months) and surface antigen levels on CD45^+^ leukocytes

Supplementary material 4 (DOCX 98 kb)

## Abbreviations:

AJCC: American Joint Committee on Cancer
DFI: disease-free interval
DMFS: distant metastasis-free survival
ECM: extracellular matrix
HR: hazard ratio
LN: lymph node
MIA: Melanoma Institute Australia
MDSC: myeloid-derived suppressor cell
NK: natural killer
OS: overall survival
TGF-β1: transforming growth factor-β1
