# Supplementary figures and images for: Surface antigen profiles of leukocytes and melanoma cells in lymph node metastases are associated with survival in AJCC stage III melanoma patients
Source: Clin Exp Metastasis. 2014 Jan 17;31(4):407–21. doi: 10.1007/s10585-014-9636-7 (PMC3973954; doi:10.1007/s10585-014-9636-7)

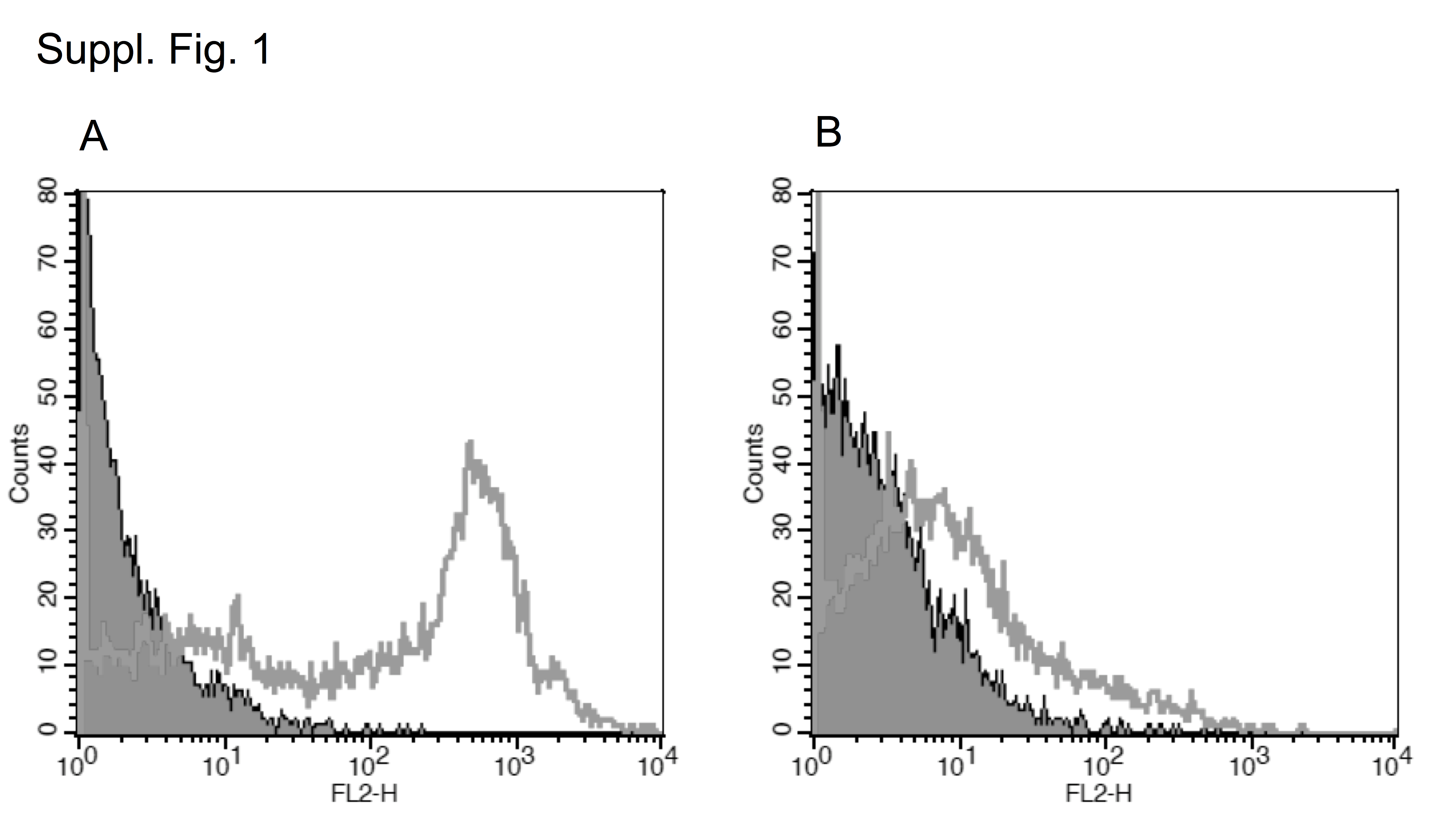

Supplement: Supplementary file 1 — To confirm the effective immuno-magnetic depletion of CD45+ leukocytes, a melanoma LN cell suspension was labelled with anti-CD45-PE or anti-IgG2a-PE and analyzed by flow cytometry. Overlays of CD45-PE and IgG2a-PE labelled cells (a) before and (b) after CD45-conjugated microbead depletion, achieved an ~ 100-fold reduction of CD45 + leukocytes [file 10585_2014_9636_MOESM1_ESM.tiff]
