# Supplementary material for: Surface antigen profiles of leukocytes and melanoma cells in lymph node metastases are associated with survival in AJCC stage III melanoma patients
Source: Clin Exp Metastasis. 2014 Jan 17;31(4):407–21. doi: 10.1007/s10585-014-9636-7 (PMC3973954; doi:10.1007/s10585-014-9636-7)
Supplement: Supplementary file 4 — Supplementary material 4 (DOCX 98 kb) [file 10585_2014_9636_MOESM4_ESM.docx]

**Supplementary Table 1: Microarray and Western blot analyses**

| *No.* | *Mel array ^a^* | *Leuk array ^b^* | *West blot ^c^* | *DMFS ^d^* | *OS ^e^* | *DFI ^f^* |
| --- | --- | --- | --- | --- | --- | --- |
| 1 | * | * |  | y | y | y |
| 2 | * | * |  | y | y | y |
| 3 | * | * |  | y | y | y |
| 4 | * | * | # | y | y | y |
| 5 | * | * |  | y | y | **n** |
| 6 | * | * | # | y | y | y |
| 7 | * | * |  | y | y | **n** |
| 8 | * | * |  | y | y | **n** |
| 9 | * | * | # | y | y | **n** |
| 10 | * | * |  | y | y | y |
| 11 | * | * | # | y | y | **n** |
| 12 | * | * | # | y | y | y |
| 13 | * | * | # | y | y | y |
| 14 | * | * |  | y | y | y |
| 15 | * | * |  | y | y | y |
| 16 | * | * |  | y | y | y |
| 17 |  | * | # | y | y | y |
| 18 | * |  |  | y | y | **n** |
| 19 | * | * | # | y | y | y |
| 20 | * |  |  | **n** | **n** | y |
| 21 | * | * | # | y | y | y |
| 22 | * | * | # | y | y | y |
| 23 | * |  |  | y | y | y |
| 24 | * | * |  | y | y | y |
| 25 | * | * |  | y | y | y |
| 26 | * | * | # | y | y | y |
| 27 | * | * |  | y | y | y |
| 28 |  |  | # | y | y | y |
| 29 |  |  | # | y | y | y |

**a** CD45^-^ enriched melanoma cells on the microarray, ‘*’ denotes successful cell binding.

**b** CD45^+^ leukocytes on the microarray, ‘*’ denotes successful cell binding.

**c** Western blot analysis of CD55 levels in CD45^+^ leukocytes, ‘#’ denotes inclusion of patient sample in analysis.

**d** Distant Metastasis Free Survival (DMFS) analysis. ‘y’, patients included in the analysis, shaded ‘y’ indicates censored patients with no distant metastasis at last follow-up or who died of other causes; ‘**n**’, patient #20 not included as was stage IV at lymph node recurrence.

**e** Overall Survival (OS) analysis. ‘y’, patients included the analysis, shaded ‘y’ indicates censored patient who died of other causes; ‘**n**’, patient #20 not included as was stage IV at lymph node recurrence.

**f** Disease Free Interval (DFI). ‘**n**’, denotes patients who were removed from this correlation analysis due to occult primary disease or the resected lymph node metastasis was not the first recurrence.
